# Insights and Implications for Health Departments From the Evaluation of New York City’s Regulations on Nutrition, Physical Activity, and Screen Time in Child Care Centers

**DOI:** 10.5888/pcd11.130429

**Published:** 2014-10-16

**Authors:** Cathy Nonas, Lynn D. Silver, Laura Kettel Khan

**Affiliations:** Author Affiliations: Lynn D. Silver, Public Health Institute, Oakland, California; Laura Kettel Khan, Centers for Disease Control and Prevention, Atlanta, Georgia.

## Abstract

In 2006, the New York City Department of Health and Mental Hygiene, seeking to address the epidemic of childhood obesity, issued new regulations on beverages, physical activity, and screen time in group child care centers. An evaluation was conducted to identify characteristics of New York City child care centers that have implemented these regulations and to examine how varying degrees of implementation affected children’s behaviors. This article discusses results of this evaluation and how findings can be useful for other public health agencies. Knowing the characteristics of centers that are more likely to comply can help other jurisdictions identify centers that may need additional support and training. Results indicated that compliance may improve when rules established by governing agencies, national standards, and local regulatory bodies are complementary or additive. Therefore, the establishment of clear standards for obesity prevention for child care providers can be a significant public health achievement.

## Background

In 2006, New York City, seeking to address factors contributing to rising rates of childhood obesity, promulgated health code regulations for group child care centers on beverages, physical activity, and screen time. Although the regulations were grounded in scientific evidence, until now, no large-scale assessment of the effect of such regulations has been conducted.

It is interesting to examine the regulations, approved in 2006 and executed in 2007, from the vantage point of 2014. Much has changed. Since 2007, new Institute of Medicine guidelines were released that advocate similar guidelines for early child care centers (1). The federal Child and Adult Care Food Program (CACFP), in which 86% of the centers in our evaluation participated, adopted guidelines similar to the New York City regulations in 2009 (2). And *Caring for Our Children*, the 3rd edition of child care standards, released in 2010 by the American Academy of Pediatrics, the American Public Health Association, and the National Resource Center for Health and Safety in Child Care and Early Education (3), reduced the amount of juice per day to be given and recommended 1% or skim milk for children aged 2 years or older. This redundancy, in which a policy that is shown or expected to change behavior is reinforced by another, may be particularly important in health policy. Although one policy may help to improve health, greater consistency among the policies of regulators, payors, and expert bodies that affect the same population may have synergistic effects.

The articles in this collection present the findings of the multimethod evaluation of the impact of the 2006 regulations. The first data collection in late 2009 included 176 child care centers, and the second data collection included 110 of the original centers 6 months later. The centers were located in high-poverty neighborhoods in all 5 boroughs of the city. Most of the children were Hispanic or non-Hispanic black, aged 3 or 4 years. The first data collection (the Center Component) was for an evaluation at the center level and included interviews with the staff and direct observation of center-level characteristics, such as whether there was physical activity in the classroom curriculum plan or low-fat milk in the refrigerator. The second data collection (the Class Component) was for an evaluation at the classroom and child level and included direct observation of the classroom staff and child behavior, such as whether children drank water or were physically active for 60 minutes each day. A detailed description of the design and methods can be found elsewhere (3).

## Optimizing Compliance

The articles in this collection show both the difficulties and ease with which centers complied with various components of the change in health code regulations. These data are important for developing strategies to optimize compliance. Although some centers had more difficulty with compliance than others, this evaluation demonstrated that most centers were able to comply with most of the changes in the regulation, suggesting that a public policy to change nutrition and physical activity through regulation can be implemented. Additionally, the data will help us identify the types of centers that should be targeted for training and technical assistance. For example, a center that lacks dedicated outdoor space may be challenged by the physical activity regulation, but by training staff in implementing an in-classroom physical activity curriculum, compliance may be improved.

## Insights From the Use of Various Methods

For policy assessment, both process and outcome evaluations are beneficial. First, we needed to assess whether center administrators and staff were aware of the new policy. Next, we needed to assess whether the centers were complying with and implementing the regulations. And finally, we needed to know if the implemented policy had the desired effect on staff and child behavior. Increased physical activity and improved nutrition, regardless of whether they ultimately affect rates of obesity, are positive health outcomes in and of themselves because they improve other aspects of health, such as cardiovascular health.

As part of the center component of the evaluation, trained data collectors interviewed staff to assess knowledge and self-reported compliance with the regulations (3). The data collected suggested that the Department of Health and Mental Hygiene (DOHMH) did a good job of disseminating information about the regulations and that center staff considered themselves to be highly compliant, self-reporting ranges of compliance with the regulations from 69% for type of juice served to 100% for television time permitted and 87% for physical activity time required (4).

In addition to examining compliance through self-report in the center component, we examined compliance more objectively through observation in the class component. We found that staff self-reports and class observation were similar for screen time and most beverage regulations but varied significantly for other items, particularly physical activity. Through direct observation, most centers were found to comply with the regulation on serving 100% juice (84.5%) and permitting 60 or fewer minutes of television time (89%). Only 26% of observed centers offered 60 minutes of physical activity per day — although it should be noted that among centers that did not meet physical activity requirements, many came close.

Observation is expensive and time intensive. Data collectors had to be trained on how to observe classrooms and child behaviors and understand the nuances, including how to accurately record portions and methods of food preparation and describe structured and unstructured activity. One rationale for observation may be simply to know how closely a center’s compliance compares with the staff’s perception of compliance. Our observations indicated some surprising results, such as incomplete compliance with the provision of water and use of only 100% juice, 2 regulations that may be considered easy to implement. Direct observation, therefore, can detect important details for understanding the full depth of compliance and the impact of these regulations on children. 

## The Importance of Child Care Center Characteristics

Most of the child care centers in our evaluation qualified for and participated in CACFP, a federal program supervised by states to reimburse the cost of meals and snacks to centers that provide food to low-income residents. Many centers did not have dedicated outdoor space or extra indoor play space, and all families of the children enrolled in the centers studied were eligible for food stamps.

When considering whether a regulation can be implemented, child care center characteristics are important. Fewer hours of operation per day, a lower student-to-teacher ratio, greater stability of staff tenure, and a better-educated staff were all associated with compliance. Longer hours and higher staff turnover were associated with lower compliance, whereas participation in CACFP or Head Start enhanced compliance with several regulations. Lower compliance rates may be due to the lack of technical assistance, guidance, or regular reminders typically provided by CACFP and Head Start.

Identifying center characteristics associated with noncompliance can help health departments or other agencies better target resources for training and technical assistance. Our evaluation suggests the importance of directing resources to centers that are not part of Head Start and do not participate in CACFP but are located in low-income neighborhoods.

## Lessons Learned About Compliance With Each Regulation

### Water

The 2006 New York City regulations state that “water should be available and easily accessible to children throughout the day, including at meals.” Eighty six percent of centers reported compliance with the water regulation in the center component (when compliance was assessed through self-report during staff interviews), and 56% of centers were compliant in the class component (when compliance was assessed through direct observation in the classroom). The difference in compliance may indicate that the wording of a regulation is open to interpretation. Words such as “available and easily accessible” may not be specific enough. For example, is a water fountain down the hall available and easily accessible? Breck et al (3) reported that centers in which water was available “down the hall” had lower water-consumption scores than centers in which water was available at the food table. The concepts of availability and accessibility are open to too much interpretation and are perhaps confounded by CACFP guidelines, which express concern that water not displace milk at meals. The lack of clarity has led to confusion in some centers. So what did we learn? That the regulation on water was not written in more specific terms created difficulty in evaluating compliance: the researchers and the centers may have interpreted the regulations differently. If the regulation had stated distinctly that water must be in a pitcher or prepoured in cups or centrally located and at the food table, the terms of compliance would have been clearer to the centers and easier to measure for the researchers. Jurisdictions in which regulations are written in more concrete terms could clarify this issue. Furthermore, standards (eg, CACFP standards) that may be perceived as not aligned with the New York City regulations may have led to reduced compliance in some centers.

### 100% Juice

The evaluation of compliance with the juice regulation was divided into 2 parts. First, if the beverage examined was not 100% juice, it was counted as a sugar-sweetened beverage and therefore found noncompliant with the juice requirement. Second, if the portion of juice was more than 6 ounces, then the center was recorded as noncompliant with the regulated portion size. Some centers were confused about whether a beverage qualified as 100% juice, suggesting the need for more nutrition education, especially in reading food and beverage labels. For example, some centers mistakenly equated the labels “100% vitamin C” with “100% juice.” During observation, 67% of centers were compliant for serving size. This finding brought to light the mealtime practice of “free pour”: teaching a child to pour his or her own drink. In centers where free pour was a learning tool, compliance with the 6-ounce juice serving per day may have been lower, but we did not evaluate the effect of free pour. Again, compliance with this regulation may have been greater had it been written with greater clarity. For example, the policy could have stated “Label must say 100% juice, and no more than 6 ounces should be prepoured into cups once per day. Centers that practice ‘free pour’ should not use juice as an allowable free-pour beverage.”

### Sugar-sweetened beverages

The level of compliance with the restriction on sugar-sweetened beverages was high, particularly for centers that were also compliant with the 100% juice requirement. In both the center and class components of the evaluation, 80% to 85% of centers were compliant. Some noncompliance may be explained by our categorizing non-100% juices as sugar-sweetened beverages and the confusion in some centers about identification of 100% juice. Therefore, more education on reading beverage labels and on what constitutes a sugar-sweetened beverage may be necessary to increase compliance. 

### Milk

Compliance with the regulation on low-fat milk for children older than 2 years was on average 90% for all centers in both the center and class components of the evaluation, but the likelihood of compliance was particularly high for Head Start centers (odds ratio, 2.85 for Head Start centers vs other centers.). There may be better understanding of different types of milk than of sugar-sweetened beverages or 100% juice. The high rate of compliance indicates the potential impact that local regulations have on compliance when they are reinforced by governing agencies and programs such as CACFP, the US Department of Agriculture, and the Special Supplemental Nutrition Program for Women, Infants, and Children.

### Screen time

The level of compliance with the regulation on screen time — 60 or fewer minutes per day for children aged 2 to5 years — was high in both the center and class components of the evaluation, as was adherence to the regulation that requires television programming to be either educational programming or programming that actively engages children in movement. Minutes of screen time did not predict sedentary activity among children. Because compliance with the screen time regulation was high, it may be feasible to align the regulation more closely with recent recommendations of only 30 minutes per week of educational screen time (5).

### Physical activity

The physical activity regulation requires children aged 12 months or older to participate in 60 minutes of physical activity per day, regardless of weather; for children aged 3 years or older, 30 minutes of that time must be structured. The level of compliance with the number of minutes of physical activity was much lower when evaluated through direct observation than by self-report. In the center component, 77% of center staff interviewed reported they met the 30 minutes of structured activity, and 86% reported they met the 60 minutes of total physical activity. In the class component, when minutes of physical activity were observed, 30% were compliant with the 30-minute requirement for structured activity and 26% reached the 60-minute requirement.

There are many reasons why the physical activity requirement is challenging to implement. Early child care centers in dense, urban settings like New York City often lack adequate space for physical activity or may have safety concerns on the playground (6,7). Additionally, structured physical activity requires that teachers demonstrate and participate in the activities. Because more than one-third of adults are sedentary (8), some teachers in child care centers may find a physical activity requirement challenging (9).

What are the characteristics that influence a center’s ability to comply with the physical activity requirement? More hours of operation and more children per class were inversely associated with compliance, suggesting that centers with long operating hours and large classroom sizes may need additional support to facilitate compliance. Dedicated outdoor space was associated with higher levels of moderate-to-vigorous activity when measured by accelerometer; this finding has been reported elsewhere (10–12). Compliant centers also used curriculum plans more often than noncompliant centers, totaling minutes of physical activity well beyond the requirement: approximately 50 minutes for structured activity, and more than 95 minutes for total physical activity.

It should be noted, though, that even in centers not complying fully with the physical activity requirement, children were physically active. On both days of observation, noncompliant centers provided approximately 15 minutes of the 30 minutes of structured activity required and approximately 40 of the 60 minutes of total physical activity required. These encouraging results suggest that with some assistance, these centers could achieve full compliance. Further evaluations might consider incorporating an additional measure such as duration of activity.

Staff training was significantly associated with meeting the physical activity requirements. Each additional teacher who participated in a 1-day SPARK! Early Childhood training (a curriculum for in-classroom physical activity) increased the likelihood of compliance by about 9%. Additional physical activity-related training (beyond SPARK!) further increased a center’s likelihood of compliance. However, training is expensive and may not be financially feasible because of ever-shrinking budgets. One possible strategy is to allocate training funds to centers most in need — for example, centers that lack dedicated outdoor space.

DOHMH has district public health offices in 3 communities that have exceptionally high poverty rates and high rates of chronic disease. Centers in these 3 communities received additional on-site technical assistance in implementing the physical activity requirements. Trainers visited each center multiple times, watching how teachers implemented the activities, helping teachers improve their technique, and ensuring that centers had sufficient equipment. Interestingly, post-training technical assistance seemed to have little additional effect. Again, an important consideration in policy design is how best to invest financial and staff resources. Allocations of money for training and other technical assistance may be helpful, but methods should be piloted and evaluated to increase the likelihood of success.

Although training clearly increased the likelihood of compliance, enacting a policy in the absence of training resources may still be beneficial. Setting clear standards to improve nutrition and increase physical activity in early child care centers will likely lead to change, however incremental. Better equipped centers will more easily comply, but even those facing challenges will likely strive to comply.

## Conclusion

The changes in the New York City health code to improve nutrition, increase physical activity and reduce screen time were largely implemented by the city’s early child care centers, although some centers were better able to fully comply than others. Other jurisdictions should consider setting standards in early child care to help improve the health of young children.
